# Highly nonlinear magnetoelectric effect in buckled-honeycomb antiferromagnetic Co_4_Ta_2_O_9_

**DOI:** 10.1038/s41598-020-69117-5

**Published:** 2020-07-23

**Authors:** Nara Lee, Dong Gun Oh, Sungkyun Choi, Jae Young Moon, Jong Hyuk Kim, Hyun Jun Shin, Kwanghyo Son, Jürgen Nuss, Valery Kiryukhin, Young Jai Choi

**Affiliations:** 1 Department of Physics, Yonsei University, Seoul, 03722 Korea; 2 Max Planck Institute for Solid State Research, Heisenbergstrasse 1, 70569 Stuttgart, Germany; 3 Department of Physics and Astronomy, Rutgers University, Piscataway, NJ 08854 USA; 4 Department of Modern Magnetic Systems, Max Planck Institute for Intelligent Systems, Heisenbergstrasse 3, 70569 Stuttgart, Germany

**Keywords:** Physics, Condensed-matter physics, Ferroelectrics and multiferroics

## Abstract

Strongly correlated materials with multiple order parameters provide unique insights into the fundamental interactions in condensed matter systems and present opportunities for innovative technological applications. A class of antiferromagnetic honeycomb lattices compounds, A_4_B_2_O_9_ (A = Co, Fe, Mn; B = Nb, Ta), have been explored owing to the occurrence of linear magnetoelectricity. From our investigation of magnetoelectricity on single crystalline Co_4_Ta_2_O_9_, we discovered strongly nonlinear and antisymmetric magnetoelectric behavior above the spin-flop transition for magnetic fields applied along two orthogonal in-plane directions. This observation suggests that two types of inequivalent Co^2+^ sublattices generate magnetic-field-dependent ferroelectric polarization with opposite signs. The results motivate fundamental and applied research on the intriguing magnetoelectric characteristics of these buckled-honeycomb lattice materials.

## Introduction

The emergence of novel cross-coupling effects generated by multiple order parameters in a wide range of materials has provided new perspectives into the interactions that occur in condensed matter systems^1,2^. Prominent examples are magnetoelectric and multiferroic materials where the cross-coupling between electric and magnetic properties has driven intense research to explore fundamental mechanisms responsible for the intrinsic magnetoelectric effects^3–9^. The primary focus of research activity in this field has on the emergence of ferroelectricity from different types of exotic magnetic orders and its dependence on applied magnetic fields. Some studies have emphasized also the potential of these materials in applications such as magnetoelectric memory and sensors by engineering their cross-coupling effects^10–13^. Despite the fact that quite a few magnetoelectric or multiferroic materials are known to us, it is still desired to discover new materials with stronger magnetoelectric coupling for enhancing the feasibility of utilizing their functionalities in device applications.

Materials composed of two-dimensional honeycomb lattices have been investigated due to possible occurrence of intriguing physical phenomena such as quantum spin liquid state^14–16^ and electronic state with Dirac-like linear dispersion^17–19^. The antiferromagnet of Co_4_Nb_2_O_9_ has recently been in focus for its linear magnetoelectric behavior^6,7,20–22^. Co_4_Nb_2_O_9_ crystallizes in a trigonal *P*$\overline{3}$*c*1 structure with two different types of honeycomb layers stacked alternately along the *c* axis. In the single crystalline Co_4_Nb_2_O_9_, grown by a floating zone method^23^, antiferromagnetic order sets in below *T*_N_ ≈ 27 K, concurrently with a linear magnetoelectric effect in applied magnetic fields^24–26^. A magnetic structure was observed as lowered monoclinic symmetry^21^ and the presence of off-diagonal elements in the magnetoelectric tensor suggests the formation of toroidal moments^27–29^.

Further studies of the magnetoelectric effect in honeycomb lattices were done on the isostructural compound Co_4_Ta_2_O_9_ (CTO)^6,30,31^. In CTO, the antiferromagnetic order emerges at *T*_N_ ≈ 20 K, simultaneously with the appearance of a dielectric anomaly and a ferroelectric polarization in applied magnetic fields. Until now, it has been believed that below *T*_N_, the ferroelectric polarization in CTO increases monotonously under increasing applied magnetic fields, similar to that in Co_4_Nb_2_O_9_^20–22^. However, these studies were performed only on polycrystalline samples, in which the physical properties are averaged out over all spatial directions due to a large number of grains of varying orientations. To overcome this challenge, we grew single crystals of CTO by utilizing the conventional flux method^32^. Despite an antiferromagnetic order of CTO on buckled-honeycomb lattices, similar to the magnetic structure of Co_4_Nb_2_O_9_^21^, the single crystalline CTO reveals strongly nonlinear magnetoelectric effect which is unique among A_4_B_2_O_9_ (A = Co, Fe, Mn and B = Nb, Ta) compounds^20,30,33–36^. This suggests the existence of two different polarization components originating from inequivalent Co^2+^ sublattices. Our nontrivial discovery calls for further experimental and theoretical studies to reveal the underlying microscopic mechanism.

## Results and discussion

**Figure 1:**
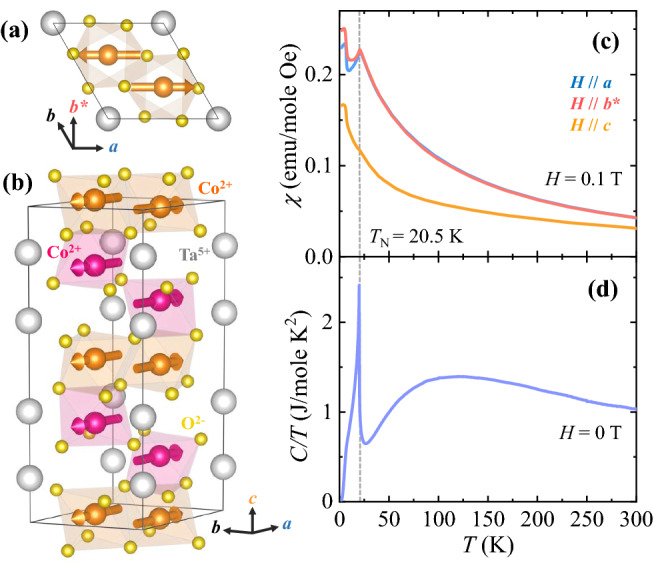
Crystallographic structure and temperature (*T*) dependence of magnetic properties. (**a**,**b**) Views of the crystal structure of Co_4_Ta_2_O_9_ (a space group *P*$\overline{3}$*c*1, No. 165) with the Co^2+^ moments in a selected magnetic domain from the top (**a**) and the side (**b**). Orange and pink spheres with arrows represent two inequivalent Co^2+^ ions and their spin directions, and light grey and yellow spheres denote nonmagnetic Ta^5+^ and O^2−^ ions, respectively. The grey box with the rhombic cross-section represents the crystallographic unit cell. (**c**) *T* dependence of magnetic susceptibility, *χ* = *M/H*, at magnetic field *H* = 0.1 T applied along the three inequivalent crystallographic orientations *a*, b^*^ and *c*. (**d**) *T* dependence of specific heat divided by the temperature, *C/T*, measured at *H* = 0 T. A dashed grey line indicates the Néel temperature, *T*_N_ = 20.5 K.

CTO crystallizes in a trigonal *P*$\overline{3}$*c*1 structure with unit cell dimensions of *a* = 0.517 nm, and *c* = 1.413 nm, obtained from the single crystal X-ray diffraction experiment (see Supplementary Information S1 for details). The crystallographic structures viewed from the top and side are depicted in Fig. 1a,b, respectively. Two dissimilar types of honeycomb layers are stacked alternatingly along the *c* axis. One layer consists of six edge-shared CoO_6_ octahedra in the same plane, while the other consists of corner-shared octahedra buckled in a zig-zag arrangement around the ring^20^. Recent neutron diffraction measurements on single crystals of CTO^37^ reveal a consistent result with the magnetic order shown in Fig. 1a,b when assuming a collinear arrangement of Co^2+^ moments. Considering the centrosymmetric trigonal structure with three-fold rotational symmetry about the *c* axis combined with two types of 180°-oriented antiferromagnetic domains leads to the possible formation of six types of 60°-oriented antiferromagnetic domains.

To examine the magnetic properties of CTO, the *T* dependence of the magnetic susceptibility, *χ* = *M/H*, was measured at *H* = 0.1 T upon warming after zero-field-cooling. The anisotropic *χ*, obtained for the *H* along the three distinguishable axes *a*, b*, and *c*, are shown in Fig. 1c. For the two orthogonal in-plane orientations, *a* and b*, the *χ* exhibits a sharp anomaly at *T*_N_ ≈ 20.5 K, indicating the emergence of antiferromagnetic order. The *T* dependence of *C/T* measured at zero *H* also shows a distinct anomaly at *T*_N_ (Fig. 1d). Above *T*_N_, the *χ* for the two in-plane orientations decreases smoothly with *T* with nearly identical shapes. On the other hand, a weak anomaly at *T*_N_ is observed in the *χ* for the *c* axis.

The overall *T* dependence of *χ*, compared between in-plane and out-of-plane orientations, shows strong magnetic anisotropy, suggesting the in-plane antiferromagnetic alignment of Co^2+^ spins. The shape of *χ* curve for *a* and b* axes are different below *T*_N_ and the faster decrease of *χ* for the *a* axis upon lowering *T* is observed because the spins in two types of the antiferromagnetic domains align along this axis. As *T* is further decreased, a sudden increase of *χ* occurs at *T*_C_ = 6.5 K. The characteristics of this transition were investigated in detail by AC *χ* measurement, which indicates the formation of a new phase such as a weakly ferromagnetic or/and glass state (see Supplementary Information S2 for details).

**Figure 2:**
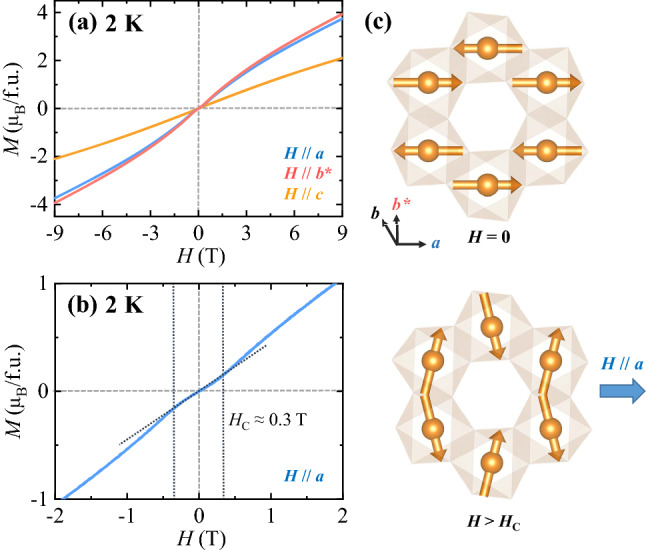
Anisotropic isothermal magnetization for Co_4_Ta_2_O_9_. (**a**) Isothermal magnetization, *M*, measured at 2 K in the *H* range of ± 9 T along the *a*, b* and *c* axes. (**b**) Magnified plot of *M* for the *a* direction. Short-dotted vertical lines indicate spin-flop transitions occurring at *H*_C_ ≈ ± 0.3 T. (**c**) Antiferromagnetic spin structure of Co^2+^ ions at *H* = 0 T (Top). Magnetic structure of Co^2+^ moments above the spin-flop transition, *H* > *H*_C_ along the *a* axis (bottom).

The isothermal *M* for the three inequivalent orientations was measured up to ± 9 T at *T* = 2 K, as shown in Fig. 2a. The *M* along the *a* direction (*M*_*a*_) shows a broad bending at a low *H* regime. Upon increasing *H* further, the *M*_*a*_ increases monotonously and reaches 3.7 μ_B_/f.u. at 9 T. The *M*_b*_ exhibits a similar *H* dependence to the *M*_*a*_; however, the magnetic moment at 9 T is found to be ~ 3.9 μ_B_/f.u., which is slightly larger than that of the *M*_*a*_. As manifested as the change in slope shown in the magnified plot of the *M*_*a*_ (Fig. 2b), the spin-flop transition occurs at *H*_C_ ≈ 0.3 T for an applied field along both *a* and b* axes due to the angular distribution of antiferromagnetic domains. The spin structures below and above the spin-flop transition are displayed in Fig. 2c. Note that this result is different from the previous results on polycrystalline samples, where the spin-flop transition occurs at a higher *H* of ~ 0.9 T, possibly due to the averaging effect over grain orientations^31^. On the other hand, the *M*_*c*_ increases almost linearly up to 9 T resulting in a magnetic moment of ~ 2.1 μ_B_/f.u. at 9 T, consistent with the strong magnetic anisotropy observed in the *T* dependence of anisotropic *χ* (Fig. 1c).

**Figure 3:**
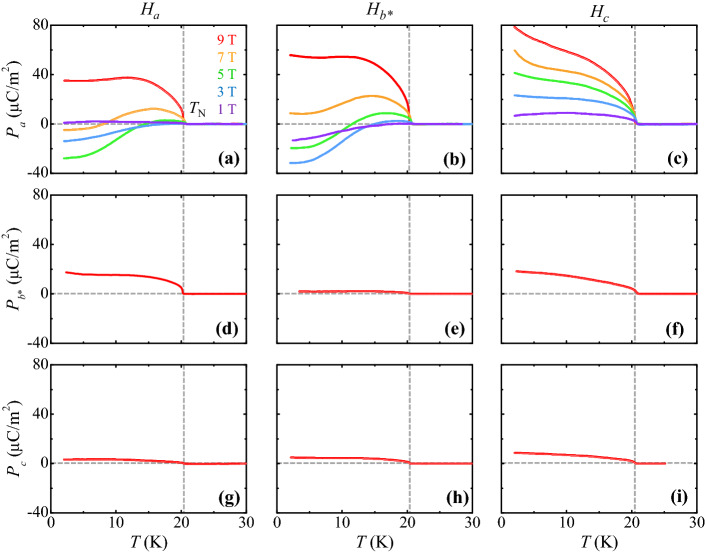
Temperature dependence of the anisotropic ferroelectric polarization. (**a**–**c**) *T* dependence of *P*_*a*_ obtained by integrating the pyroelectric current after poling from 100 to 2 K in at *H*_*a*_, *H*_b*_, and *H*_*c*_, respectively. *P*_*a*_ was measured at *H* = 1, 3, 5, 7 and 9 T. (**d**–**f**) *T* dependence of *P*_b*_ measured at *H*_*a*_ = 9 T, *H*_b*_, and *H*_*c*_, respectively. (**g**–**i**) *T* dependence of *P*_*c*_ measured at *H*_*a*_ = 9 T, *H*_b*_, and *H*_*c*_, respectively.

The anisotropic characteristics of magnetoelectric properties were examined through the *T* dependence of *P* for the *a*, b*, and *c* axes. The magnitude of *P* was obtained by integrating the pyroelectric current density measured after poling in an electric field along the direction of *P* and *H* up to 9 T for the three different orientations, as shown in Fig. 3. Interestingly, the *P* emerges dominantly along the *a* axis below *T*_*N*_ (*P*_*a*_, Fig. 3a–c) with an unusual *T* dependence upon increasing *H*. The other components of *P* do not vanish (*P*_b*_ and *P*_*c*_, Fig. 3d–i) similar to the *T* dependence of *P* in Co_4_Nb_2_O_9_^21^. In detail, Fig. 3b shows the *T*-dependence of *P*_*a*_ at *H* = 1, 3, 5, 7, and 9 T along the b* axis (*H*_b*_). The *P*_*a*_ at *H*_b*_ = 1 T starts from a negative value of − 13.2 μC/m^2^ at 2 K, increases monotonously to zero upon increasing *T*, and disappears at *T*_*N*._ At *H*_b*_ = 3 T, *P*_*a*_ exhibits the largest negative value of − 32.2 μC/m^2^ at 2 K and crosses zero *P*_*a*_ at approximately 15 K. A similar trend of change in the sign of *P*_*a*_ is observed at *H*_b*_ = 5 T with an upward shift in the overall magnitude of *P*_*a*_. The *P*_*a*_ at *H*_b*_ = 7 and 9 T retains positive values throughout the whole *T* range below *T*_*N*_, and shows its maximum magnitude of 55.9 μC/m^2^ at 2 K and *H*_b*_ = 9 T. This strongly nonlinear magnetoelectric behavior is also observed in *P*_*a*_ at different values of *H*_*a*_ (Fig. 3a). At *H*_*a*_ = 1 T, the *P*_*a*_ is very small in magnitude and shows the negligible *T* dependence. The values of *P*_*a*_ at *H*_*a*_ = 3, 5, and 7 T are all negative at low temperatures. In contrast to the case of an in-plane *H*, the *P*_*a*_ under an applied *H*_*c*_ tends to increase gradually as *H*_*c*_ is increased, maintaining a positive value throughout the entire range of *T* below *T*_*N*_ (Fig. 3c). The *P*_*a*_ at *H*_*c*_ = 9 T and 2 K is found to be 78.7 μC/m^2^ (Fig. 3c), which is approximately twice that of *P*_*a*_ = 34.9 μC/m^2^ at *H*_*a*_ = 9 T and 2 K (Fig. 3a).

**Figure 4:**
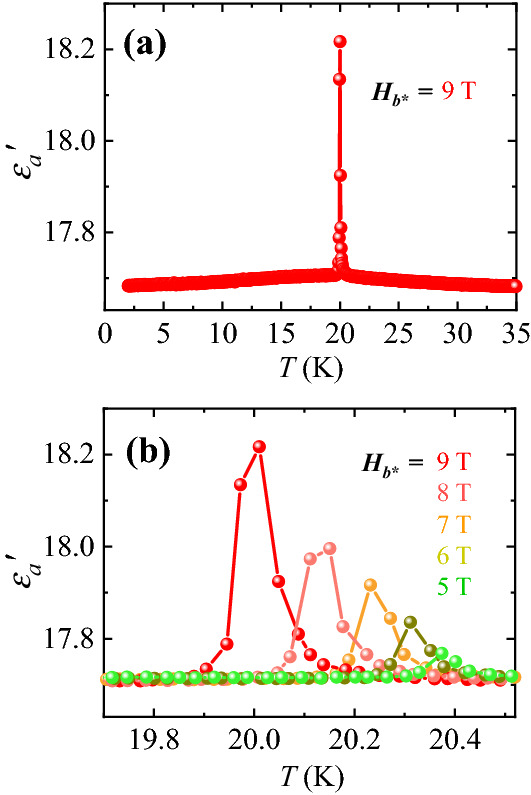
Dielectric constant along the *a* axis at *H*_b*_ with *f* = 100 kHz. (**a**) *T* dependence of dielectric constant, *ε*_*a*_*'*, below 35 K at *H*_b*_ = 9 T. (**b**) *T* dependence of *ε*_*a*_*'* in a narrow range of *T* near *T*_N_ at *H*_b*_ = 5, 6, 7, 8, and 9 T.

Figure 4a shows the *T*-dependence of dielectric constant for *E*//*a* (*ε*_*a*_*'*), measured at *H*_b*_ = 9 T and *f* = 100 kHz. The *ε*_*a*_*'* at 9 T exhibits a very sharp peak at 20.02 K with a 2.8% change in its magnitude (at the peak maximum). The sharpness of the peak at 9 T is characterized by the very small full width at half maximum (FWHM) estimated to be only 0.08 K, which indicates a good crystal quality. As *H* is decreased, the peak of *ε*_*a*_*'* shifts progressively to a higher *T* with a gradual reduction of the peak height (Fig. 4b) and almost disappears at 4 T. At 5 T, a tiny peak in *ε*_*a*_*'*, with only 0.27% change in the overall magnitude, occurs at 20.37 K.

**Figure 5:**
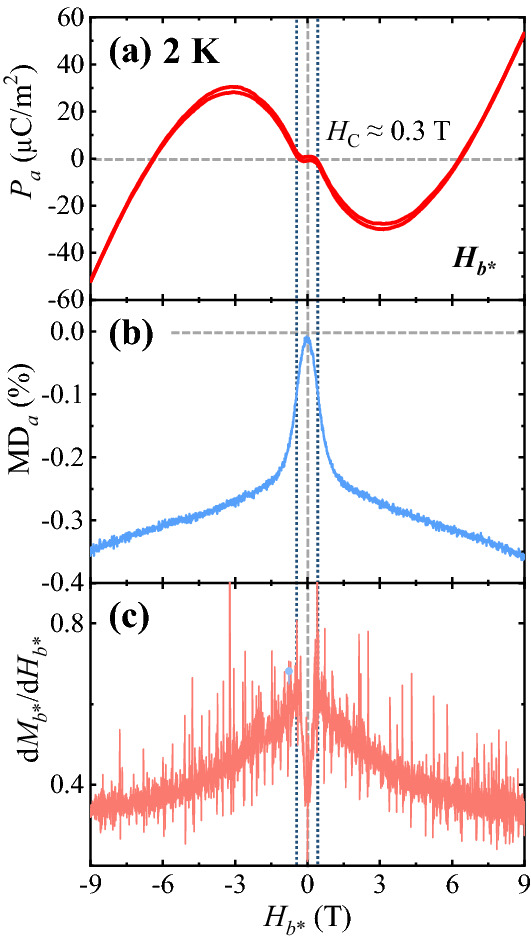
Comparison of electric and magnetic properties. (**a**) *H*_b*_ dependence of *P*_*a*_ at *T* = 2 K. (**b**) *H*_b*_ dependence of the magnetodielectric effect along the *a* axis, MD_*a*_ (%) = $\frac{{\varepsilon^{\prime}\left( H \right) - \varepsilon^{\prime}\left( {0 {\text{T}}} \right)}}{{\varepsilon^{\prime}\left( {0 {\text{T}}} \right)}} \times 100$, measured with AC excitation of *E*_*a*_ = 1 V at *f* = 100 kHz and *T* = 2 K. (**c**) *H*_b*_ derivative of *M*_b*_ at 2 K.

The nonlinear behavior of *P* and the intricate relationship between magnetic and electric properties in CTO were examined in detail by comparing the *H* dependence of *P*, *M*, and *ε'* at 2 K. The isothermal *P*_*a*_ was obtained by integrating the magnetoelectric current density, measured by sweeping the *H*_b*_ between 9 and  − 9 T at 2 K after poling in *H*_b*_ = 9 T and *E*_*a*_ = 4.72 kV/cm, as shown in Fig. 5a. Starting from the maximum value of *P*_*a*_ = 52.5 μC/m^2^ at 9 T, the *P*_*a*_ decreases upon decreasing *H*_b*_ and becomes zero at 6.3 T. As *H*_b*_ is decreased further, the *P*_*a*_ shows a broad minimum at 3.2 T with *P*_*a*_ =  − 27.5 μC/m^2^. Below *H*_C_ ≈ 0.3 T, *P*_*a*_ disappears. Further decrease in *H* in the negative direction leads to the antisymmetric *H* dependence of the *P*_*a*_. The sweeping of *H*_b*_ from − 9 to + 9 T completes the isothermal *P*_*a*_ curve, showing negligible magnetic hysteresis. In Fig. 5b, the magnetodielectric (MD) effect, described by the variation of *ε*_*a*_*'* by applying *H*_b*_ and defined as MD_*a*_ (%) = $\frac{{\varepsilon^{\prime}\left( H \right) - \varepsilon^{\prime}\left( {0 {\text{T}}} \right)}}{{\varepsilon^{\prime}\left( {0 {\text{T}}} \right)}} \times 100$, was measured up to ± 9 T at *f* = 100 kHz and *T* = 2 K. The initial curve of MD_*a*_ exhibits a slight curvature at low *H*_b*_ regime and the maximum slope at *H*_C_ ≈ 0.3 T. Above *H*_C_, the MD_*a*_ reduces more gradually which becomes almost linear above *H*_b*_ = 1.5 T. The maximum variation of MD_*a*_ is found to be approximately − 0.36% at 9 T. The full MD_*a*_ curve appears to be symmetric because the direction of *P*_*a*_ is indistinguishable in the AC excitation of *E*_*a*_ for the *ε*_*a*_*'* measurement. For a precise comparison with the MD_*a*_, the *H*_b*_ derivative of isothermal *M*_b*_, d*M*_b*_/d*H*_b*_ at 2 K is also plotted in Fig. 5c. The d*M*_b*_/d*H*_b*_ increases linearly up to *H*_C_ and reveals a kink at *H*_C_, after which it begins to decrease. To elucidate the *H*_*a*_ and *H*_*c*_ dependences of *P*_*a*_ (Fig. 3a,c), the detailed field dependent behaviors as Fig. 5 are also included in the Supplementary Information S3.

**Figure 6:**
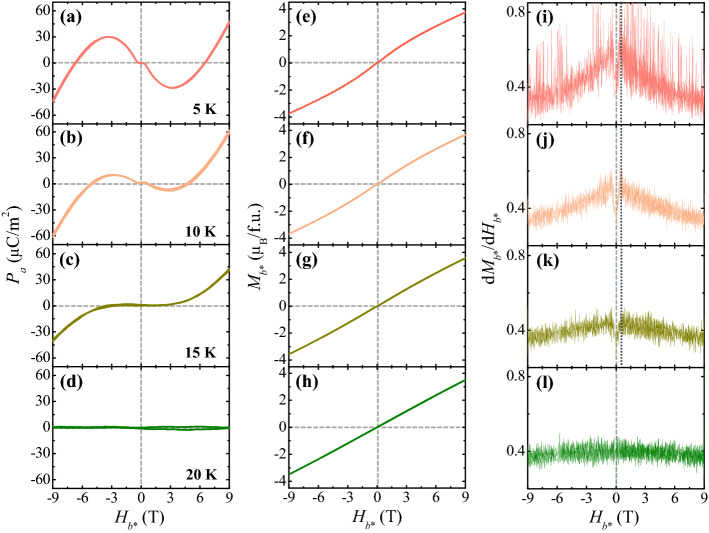
Temperature evolution of ferroelectric polarization and magnetization. (**a**–**d**) *H*_b*_ dependence of ferroelectric polarization (*P*_*a*_) at *T* = 5, 10, 15 and 20 K, respectively, obtained by integrating the magnetoelectric current measured changing *H*_b*_ at the rate of 0.01 T/s up to ± 9 T after poling in *E*_*a*_ = 4.72 kV/cm and *H*_b*_ = 9 T. (**e**–**h**) *H*_b*_ dependence of magnetization (*M*_b*_) at *T* = 5, 10, 15 and 20 K, respectively, measured up to ± 9 T. (**i**–**l**) *H*_b*_ derivative of *M*_b*_ at *T* = 5, 10, 15 and 20 K, respectively.

The *T* evolution of strongly nonlinear magnetoelectric effect in CTO is presented, which shows that the major features are preserved at 10 K above *T*_C_ = 6.5 K. Figure 6 shows the comparison among isothermal *P*_*a*_, *M*_b*_, and d*M*_b*_/d*H *_b*_ at *M*_b*_ up to ± 9 T and *T* = 5, 10, 15, and 20 K below *T*_N_. At 5 K, the overall *H*_b*_ dependences of *P*_*a*_ and *M*_b*_ tend to behave akin to those at 2 K (Figs. 2a, 5a). In comparison with the *P*_*a*_ at 2 K, the maximum value of *P*_*a*_ at 5 K and 9 T reduces slightly to 45.1 μC/m^2^ (Fig. 6a) and the *M*_b*_ at 9 T also decreases to ~ 3.72 μ_B_/f.u. (Fig. 6e). Upon decreasing *H*_b*_, a broad minimum of the *P*_*a*_ (= − 31.8 μC/m^2^) occurs at 3.1 T (Fig. 6a) and the d*M*_b*_/d*H*_b*_ at 5 K reveals kinks at *H*_C_ =  ± 0.3 T (Fig. 6i), consistent with the plateau region within *H*_C_ in the *P*_*a*_ curve (Fig. 6a). At 10 K, the broad minimum of *P*_*a*_ occurs at 2.9 T with a significantly reduced value of − 8.1 μC/m^2^ (Fig. 6b). However, the maximum value of *P*_*a*_ = 58.9 μC/m^2^ at 9 T is found to be the largest despite the slight decrease of *M*^b*^ (~ 3.64 μ_B_/f.u., Fig. 6f). At 15 K, the regime of nearly zero *P*_*a*_ extends up to ± 3.0 T with the absence of the broad minimum (Fig. 6c). At 20 K, the *P*_*a*_ almost disappears (Fig. 6d) throughout the measurement region of *H*_b*_ while the *M*_b*_ shows a linear increase upon increasing *H*_b*_ and finally becomes ~ 3.50 μ_B_/f.u. at 9 T (Fig. 6h).

Distinctive from the linear magnetoelectric behavior in the isostructural Co_4_Nb_2_O_9_, the electric polarization in CTO arises at the spin-flop transition above which strongly nonlinear and antisymmetric field dependence was observed. The linear magnetoelectric response and controllable electric polarization by rotating magnetic fields^38^ in Co_4_Nb_2_O_9_ have recently been explained by several theoretical works such as the orbital model incorporating local spin–orbit coupling at the site of Co^2+^ ion^39^ and symmetry interpretation considering local C_3_ point group^40^. In such theoretical analyses, the contributions from two types of magnetic sublattices, which are associated with two dissimilar types of honeycomb layers, to the net electric polarization are not distinguishable. Another theoretical work based on the Hartree–Fock calculations presents a noticeable consequence that each magnetic sublattice produces electric polarization with a different magnitude and direction, each of which varies linearly with the applied magnetic field strength^6^. The superposition of two different contributions leads to a linear behavior in the total polarization. However, the highly-nonlinear magnetoelectric effect of our CTO in the *P*_*a*_ under *H*_*a*_ and *H*_b*_ implies the more intricate contribution of each sublattice to the magnetic-field dependent polarization. In particular, above the spin-flop transition, the dominant negative-polarization arising from one sublattice gives rise to the negative net *P*_*a*_, but the gradual increase of the positive-polarization from the other sublattice results in the broad minimum and further increase of the net *P*_*a*_ upon increasing the field. Therefore, our results motivate more elaborate theoretical calculations comprising other factors such as additional lattice and magnetic domain contributions, and possible change of magnetic structure driven by electric field poling, which have not been considered in the previous studies.

## Conclusion

In summary, we have synthesized single crystals of magnetoelectric Co_4_Ta_2_O_9_ and explored magnetic and magnetoelectric properties along different crystallographic orientations. Despite the presence of several off-diagonal components, the dominant magnetic-field-driven change of polarization occurs for the *a* axis. More importantly, an antiferromagnetic order below *T*_N_ = 20.5 K leads to a highly nonlinear magnetoelectric effect above the spin-flop transition for in-plane magnetic fields. This is clearly different from the linear magnetoelectricity in other isostructural compounds, and indicates the complex evolution of polarization components with opposite signs originating from two different Co^2+^ sublattices. Our results provide insights into fundamental magnetoelectric interactions in the family of the buckled-honeycomb magnetoelectric magnets, paving way for the discovery of novel materials for magnetoelectric functional applications.

## Methods

Hexagonal plate-like single crystals of CTO were grown by the conventional flux method with NaF, Na_2_CO_3_, and V_2_O_5_ fluxes in air^32^. Co_3_O_4_, and Ta_2_O_5_ powders were mixed in the stoichiometric ratio and ground in a mortar, followed by pelletizing and calcining at 900 °C for 10 h in a box furnace. The calcined pellet was finely reground and sintered at 1,000 °C for 15 h. After regrinding, the same sintering procedure was carried out at 1,100 °C for 24 h. A mixture of pre-sintered polycrystalline powder and fluxes was heated to 1,280 °C in a Pt crucible. It was melted at the soaking *T*, slowly cooled to 800 °C at a rate of 1 °C/h, and cooled to room *T* at a rate of 100 °C/h.

The temperature (*T*) and magnetic-field (*H*) dependences of the DC magnetization (*M*) were measured using a vibrating sample magnetometer at *T* = 2–300 K and *H* = – 9 to 9 T in a physical properties measurement system (PPMS, Quantum Design, Inc.). The specific heat (*C*) was measured with the standard relaxation method in the PPMS. The *T* and *H* dependences of dielectric constant (*ε'*) were observed at *f* = 100 kHz using an LCR meter (E4980, Agilent). The *T* and *H* dependences of electric polarization (*P*) was obtained by integrating pyro- and magneto-electric currents, respectively, measured after poling in a static electric field (*E*).

## Supplementary information

Supplementary Information
