# Supplementary material for: Highly nonlinear magnetoelectric effect in buckled-honeycomb antiferromagnetic Co4Ta2O9
Source: Sci Rep. 2020 Jul 23;10:12362. doi: 10.1038/s41598-020-69117-5 (PMC7378256; doi:10.1038/s41598-020-69117-5)
Supplement: Supplementary file 1 — Supplementary Information [file 41598_2020_69117_MOESM1_ESM.pdf]

Supplementary Information for **Highly nonlinear magnetoelectric effect in buckled-honeycomb antiferromagnetic $Co_4Ta_2O_9$**

Nara Lee[1,*], Dong Gun Oh[1,*], Sungkyun Choi[2,3], Jae Young Moon[1], Jong Hyuk Kim[1], Hyun Jun Shin[1], Kwanghyo Son[4], Jürgen Nuss[2], Valery Kiryukhin[3] and Young Jai Choi[1]

[1]Department of Physics, Yonsei University, Seoul 03722, Korea

[2]Max Planck Institute for Solid State Research, Heisenbergstrasse 1, 70569 Stuttgart, Germany

[3]Department of Physics and Astronomy, Rutgers University, Piscataway, New Jersey 08854, USA

[4]Department of Modern Magnetic Systems, Max Planck Institute for Intelligent Systems, Heisenbergstrasse 3, D-70569 Stuttgart, Germany

*These authors contributed equally to this work.

Correspondence and requests for materials should be addressed to Y. J. C. (phylove@yonsei.ac.kr)

S1. Single-crystal X-ray diffraction on $Co_4Ta_2O_9$ (CTO)

A clean CTO crystal was selected in high viscosity oil, and was mounted on a loop made of Kapton foil (Micromounts™, MiTeGen, Ithaca, NY). Diffraction data were collected at 298 K with a SMART APEXII CCD X-ray diffractometer (Bruker AXS, Karlsruhe, Germany), using graphite-monochromated Mo-Kα radiation. The reflection intensities were integrated with the SAINT subprogram in the Bruker Suite software[1], and a multi-scan absorption correction was applied, using SADABS[2]. The structure was refined by full-matrix least-square fitting with the SHELXTL software package[3,4]. Crystallographic information and data collection details are given in Table S1, and atomic coordinates are in Table S2. Further details of the crystal structure investigations may be obtained from the Fachinformationszentrum Karlsruhe, 76344 Eggenstein-Leopoldshafen, Germany (Fax: +49-7247-808-666; E-Mail: crysdata@fiz-karlsruhe.de, http://www.fiz-karlsruhe.de/request for deposited data.html) on quoting the depository number CSD-1996769.

Table S1. Crystallographic data of $Co_4Ta_2O_9$ (CTO) as obtained from single-crystal X-ray diffraction at 298 K.

	$Co_4Ta_2O_9$
**Formula weight**	741.62
**Crystal shape, color**	block, black
**Space group (no.), *Z***	$P\bar{3}c1$ (no. 165), 2
**Lattice parameters /Å**	a = 5.1718(13)<br>c = 14.127(3)<br>c/a = 2.732
***V* /Å^3**	327.3(2)
**ρ_{xray} /g×cm^{-3}**	7.526
**Crystal size /mm^3**	0.15×0.10×0.05
**Diffractometer**	SMART APEX II, Bruker AXS
**X-ray radiation, λ/Å**	Mo-Kα, 0.71073
**Absorption correction**	Multi-scan, SADABS[2]
**2θ range /°**	5.76° ≤ 2θ ≤ 72.84°
**Index ranges**	-8 ≤ h ≤ 8<br>-8 ≤ k ≤ 8<br>-22 ≤ l ≤ 23
**Reflections collected**	5468
**Data, R_{int}**	540, 0.0409
**No. of parameters**	25
**Transmission: t_{min}, t_{max}**	0.034, 0.110
**Extinction coefficient**	0.0241(7)
**Final R indices [I > 2σ(I)]**	R1 = 0.0158, wR2 = 0.0364
**R indices (all data)**	R1 = 0.0165, wR2 = 0.0378

Table S2. Atomic coordinates and equivalent isotropic displacement factors for CTO at 298 K.

atom	site	*x*	*y*	*z*	U_{eq} (Å^2)
**Ta**	4*c*	0	0	0.14280(2)	0.00843(7)
**Co1**	4*d*	1/3	2/3	0.19186(4)	0.0103(1)
**Co2**	4*d*	1/3	2/3	–0.01396(4)	0.0100(1)
**O1**	6*f*	0	0.2870(3)	1/4	0.0102(3)
**O2**	12*g*	0.0230(3)	0.6814(3)	0.0844(1)	0.0119(2)

S2. AC magnetic susceptibility of CTO

Figure S1(a) shows the temperature (T) dependence of the DC magnetic susceptibility (χ), measured at H = 0.1 T along the b* axis (H_{b*}), on warming after zero-field-cooling (ZFC) and cooling in the same field (FC). Upon decreasing T, a sudden increase of χ occurs with the maximum slope determined by the T derivative of χ at T_C = 6.5 K. Despite the distinct anomaly of χ at T_C, only a slope change is found in the heat capacity (C) divided by the T, C/T, as shown in Fig. S1(b). This could imply the onset of a new phase below the antiferromagnetic order possibly with a weakly ferromagnetic moment or/and a spin glass state[5,6]. The T at which the ZFC and FC χ curves begin to split was observed at T_f = 5.6 K, indicative of the onset of magnetic irreversibility. To further understand the nature of low T anomalies, we did AC χ measurements. The detailed characteristics of the transition were investigated by the T dependence of real (χ') and imaginary (χ'') parts of AC susceptibility, measured along the b* axis using AC field amplitude (H_{AC}) of 8 Oe with various frequencies, f = 1, 50 and 250 Hz, and using 2 Oe with f = 1000 Hz in a magnetic properties measurement system (MPMS3, Quantum Design, Inc.). DC magnetic field of 0.1 T was also applied along the b* axis during the measurement. The measured χ' and χ'' results are shown in Figs. S1(c) and (d), respectively. At T_N, both χ' and χ'' do not depend on the oscillating frequency. Unlike this behavior, the χ' near T_f might show the f dependence as the peak seems to move with frequencies. Moreover, there is a discernible dissipative behavior seen in χ''. The irreversibility of χ between ZFC and FC, and the weak f dependence of χ' indicates the freezing of an additional glassy state below T_f.

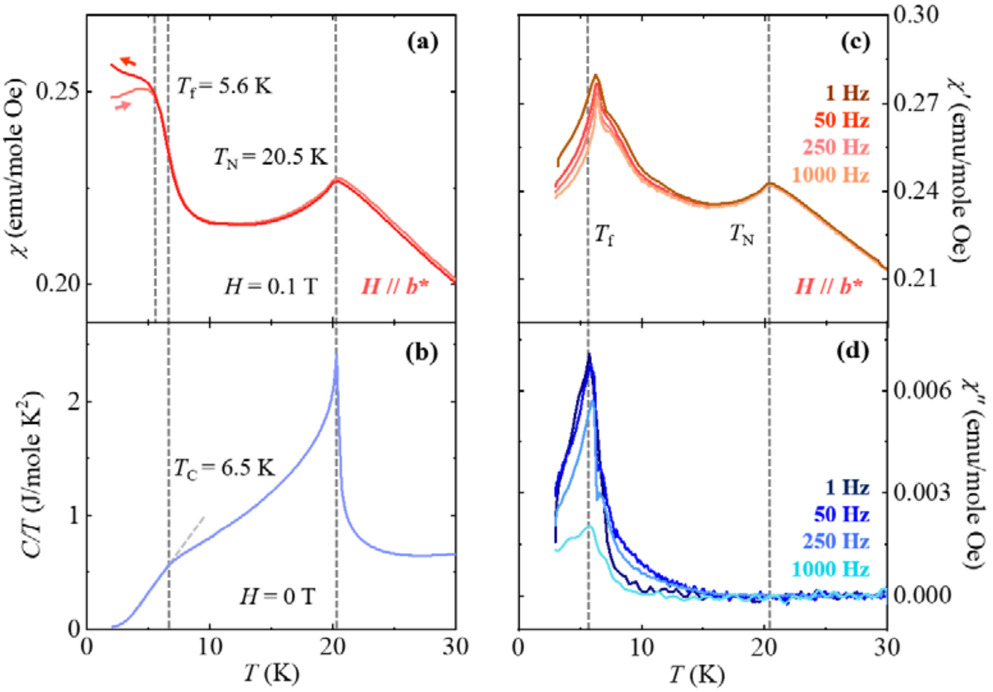

Figure S1. Comparison between DC and AC magnetic susceptibilities. (a) T dependence of the magnetic susceptibility, $\chi = M/H$, of CTO at H_{b^*} = 0.1 T, measured upon warming after zero-field cooling and upon cooling. The vertical dashed lines indicate the antiferromagnetic transition, a possible short-range weak ferromagnetic transition, and freezing temperature at T_N = 20.5 K, T_C = 6.5 K and T_f = 5.6 K, respectively. (b) T dependence of heat capacity divided by the temperature, C/T, measured in zero H up to 30 K. (c)-(d) T dependence of real (χ') and imaginary (χ'') parts of AC χ, measured at various frequencies, f = 1, 50, and 250 Hz (H_{AC} = 8 Oe) and f = 1000 Hz (H_{AC} = 2 Oe), and DC H = 0.1 T along the b* axis.

S3. The H_a dependence of P_a, MD$_a$ and dM_a/H_a, and the H_c dependence of P_a, MD$_a$ and dM_c/H_c

The isothermal P_a was obtained by integrating the magnetoelectric current density, measured by sweeping the H_a between 9 and −9 T at 2 K after poling in H_a = 9 T and E_a = 4.72 kV/cm, as shown in Fig. S2(a). The H_a dependence of P_a exhibits similar nonlinear and asymmetric behavior of the H_{b^*} dependent P_a (Fig. 5(a) of the main manuscript). In contrast to the H_{b^*} dependent MD$_a$ (Fig. 5(b) of the main manuscript), the H_a dependence of MD$_a$ appears to be positive in the entire range of H_a sweeps with the maximum variation of ~0.16 % (Fig. S2(b)).

In Fig. S2(c), the dM_a/dH_a increases linearly up to H_C and reveals a kink at H_C, after which it begins to decrease.

The T-dependence of ε_a' measured at $H_c = 9$ T also shows a very sharp peak at 20.22 K (Fig. S3(a)). The variation in the magnitude of ε_a' at the peak maximum is found to be 5.9 %, which is approximately twice as the value found for $H_{b^*} = 9$ T (Fig. 4(a) of the main manuscript). However, the FWHM is 0.08 K, which is similar to that for $H_{b^*} = 9$ T. In the zoomed data shown in Fig. S3(b), one can clearly see that the peak of ε_a' gradually moves to slightly higher T with continuous suppression of the peak height when H_c varies from 9 to 1 T. In contrast to the behavior of ε_a' at H_{b^*} (Fig. 4(b) of the main manuscript), a small peak remains even at $H_c = 1$ T. Figure S4 reveals the H_c dependences of P_a, MD_a, and dM_c/dH_c. The P_a at $H_c = 9$ T is found to be 79.1 μC/m^2 which is approximately 1.5 times the value of P_a at $H_{b^*} = 9$ T (Fig. 5(a) of the main manuscript). As the H_c is decreased from 9 T, the P_a decreases monotonously with a slight change of its slope between 6 and 3 T. Further decrease in H_c induces continuous reduction of the P_a, resulting in an antisymmetric field dependence of P_a for negative values of H_c. The MD_a, shown in Fig. S4(b), increases slightly from 9 T and a small change in slope occurs below 6 T. It decreases monotonously below 3 T, accompanied by symmetric behavior for negative H_c values, with approximately 0.4% variation in the whole measurement range of H_c. The dM_c/dH_c in Fig. S4(c) varies smoothly in the entire H_c range, dissimilar from the distinct anomaly observed at the spin-flop transition for the H_{b^*} direction.

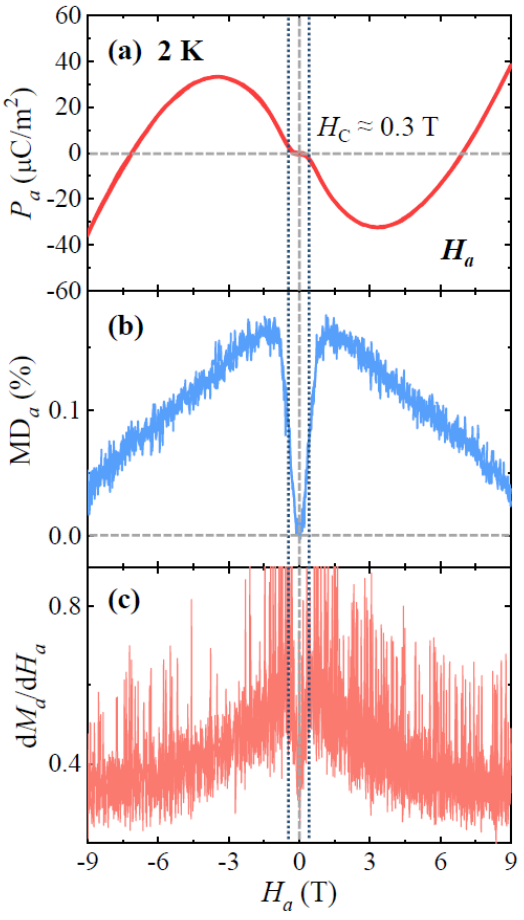

Figure S2. Comparison between electric and magnetic properties. (a) H_a dependence of P_a at $T = 2$ K. (b) H_a dependence of the magnetodielectric effect along the *a* axis, MD_a (%) = $\frac{\varepsilon'(H)-\varepsilon'(0\ \mathrm{T})}{\varepsilon'(0\ \mathrm{T})} \times 100$, measured with AC excitation of $E_a = 1$ V at $f = 100$ kHz and $T = 2$ K. (c) H_a derivative of M_a at 2 K.

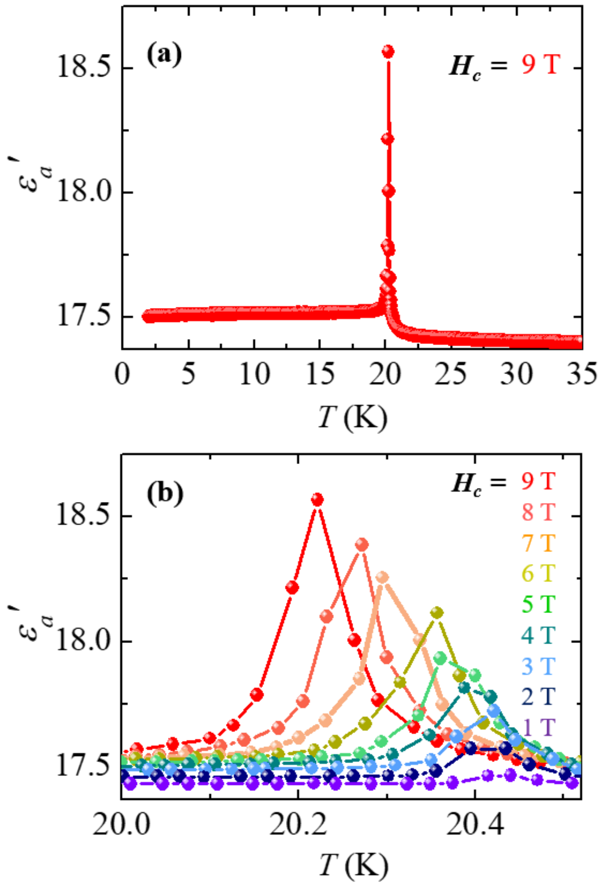

Figure S3. Dielectric constant along the *a* axis at H_c. (a) Temperature dependence of dielectric constant, ε_a', below 35 K at $H_c = 9$ T. (b) Temperature dependence of ε_a' in the narrow range of T near T_N at $H_c = 1\sim9$ T.

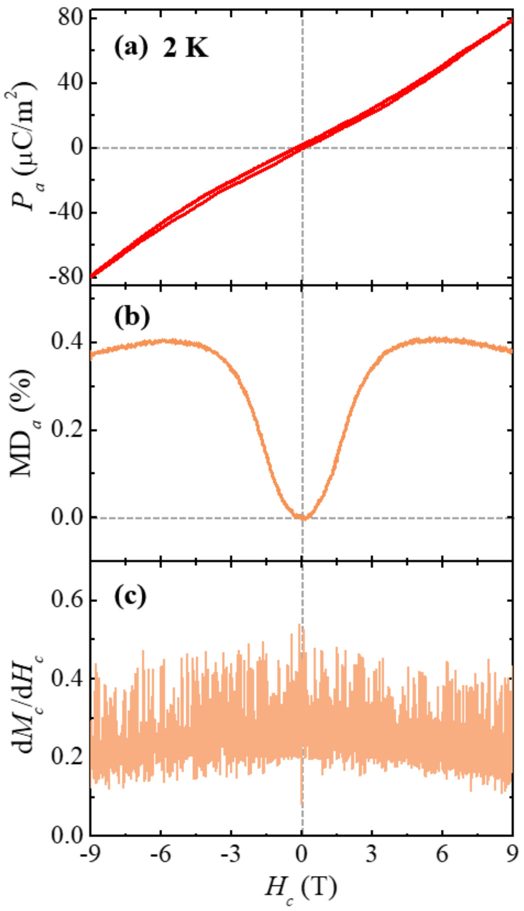

Figure S4. Comparison between electric and magnetic properties. (a) H_c dependence of P_a at $T = 2$ K. (b) H_c dependence of the magnetodielectric effect along the *a* axis, MD$_a$ (%) = $\frac{\varepsilon'(H)-\varepsilon'(0\ \mathrm{T})}{\varepsilon'(0\ \mathrm{T})} \times 100$, measured with AC excitation of $E_a = 1$ V at $f = 100$ kHz and $T = 2$ K. (c) H_c derivative of M_c at 2 K.

References

1. Bruker Suite, version 2015/9. Bruker AXS Inc., Madison, WI, **2015**.

2. Sheldrick, G. M. *SADABS — Bruker AXS area detector scaling and absorption*, version 2014/3, University of Göttingen, Germany **2014.**

3. Sheldrick, G. M. A Short History of SHELX. *Acta Crystallogr., Sect. A: Found. Crystallogr.* **64**, 112-122 (2008).

4. Sheldrick, G. M. Crystal Structure Refinement with SHELXL. *Acta Crystallogr., Sect. C:*

Struct. Chem. **71**, 3-8 (2015).

5. Wakimoto, S., Ueki, S., Endoh, Y. & Yamada, K. Systematic study of short-range antiferromagnetic order and the spin-glass state in lightly doped $La_{2-x}Sr_xCuO_4$. *Phys. Rev. B* **62**, 3547-3553 (2000).

6. Li, D. X. *et al.* Spin-glass behavior with extended short-range ferromagnetic order in U_2RhSi_3. *J. Phys.: Condens. Matter* **11**, 8263-8274 (1999).
